# Henoch-Schönlein Nephritis Manifesting with Purpura 15 years after Diagnosis of IgA Nephropathy

**DOI:** 10.1155/2019/1042648

**Published:** 2019-10-29

**Authors:** Hideaki Yamabe, Mitsuaki Kaizuka, Satoru Tsunoda, Tasuku Nagasawa, Kazuo Nomura, Michiko Shimada

**Affiliations:** ^1^Internal Medicine, EST Clinic, Hirosaki, Japan; ^2^Department of Nephrology, Hirosaki University Hospital, Hirosaki, Japan; ^3^Internal Medicine, Japanese Red Cross Ishinomaki Hospital, Ishinomaki, Japan; ^4^Aoyama Nomura Dermatology Clinic, Hirosaki, Japan

## Abstract

Henoch-Schönlein nephritis or immunoglobulin A (IgA) vasculitis is characterized by purpura, arthralgia, abdominal pain, and glomerulonephritis with glomerular IgA deposition. Notably, the presence of purpura is essential to diagnose this disease. We report the case of a patient in whom proteinuria and haematuria were detected during screening tests and he was diagnosed with IgA nephropathy at 20 years of age. Corticosteroids were administered for 7 years and were subsequently tapered. At 35 years of age, he noticed purpura on his lower extremities and was diagnosed with anaphylactoid purpura. Following the appearance of purpura, urinalysis revealed an increase in urinary protein levels from 0.7 g/g creatinine (Cr) to 1.4 g/gCr, and his serum Cr levels increased from 1.1 mg/dL to 1.35 mg/dL. Two months later purpura subsided, and his urinary protein level and serum Cr level were restored to the former levels. Although the cause remains unknown, an interval may occasionally be observed between the appearance of purpura and urinary abnormalities. However, to our knowledge to date, a 15-year interval is the longest interval, in such cases, reported in the literature.

## 1. Introduction

Henoch-Schönlein nephritis or immunoglobulin A (IgA) vasculitis (IgAV) is characterized by purpura, arthralgia, abdominal pain, and glomerulonephritis with glomerular IgA deposition. Notably, the presence of purpura is essential to establish the diagnosis in these patients [1]. Usually, patients show urinary abnormalities with a purpuric rash concomitantly or within a month. We report a patient of Henoch-Schönlein nephritis who developed purpura 15 years after the diagnosis of IgA nephropathy (IgAN). To our knowledge to date, 15 years is the longest interval between the detection of urinary abnormalities and the development of purpura.

## 2. Case Report

**Figure 1:**
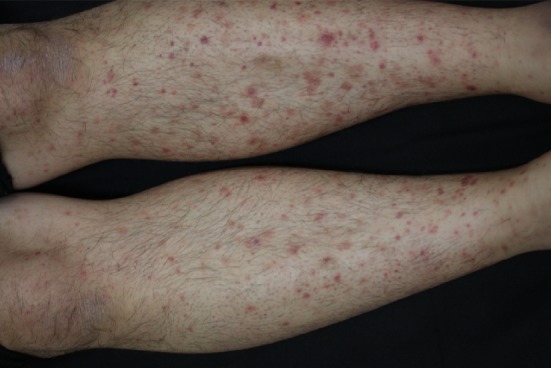
Purpuric rash on the patient's lower extremities.

**Figure 2:**
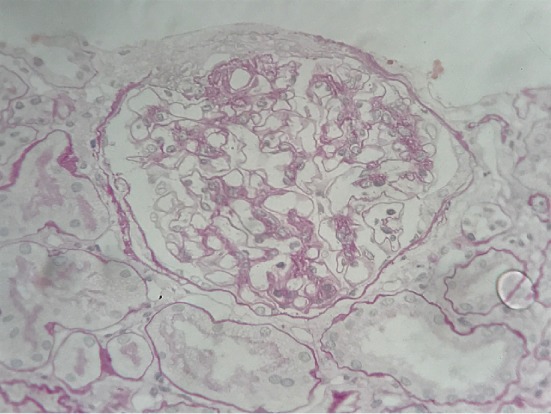
Mesangial proliferative glomerulonephritis was demonstrated (PAS stain).

**Figure 3:**
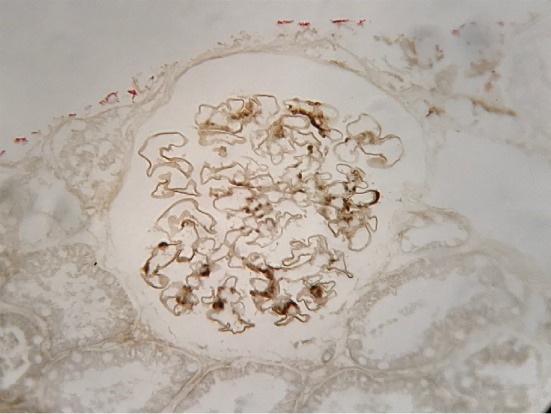
Mesangial IgA deposition was observed by immunohistochemistry.

**Figure 4:**
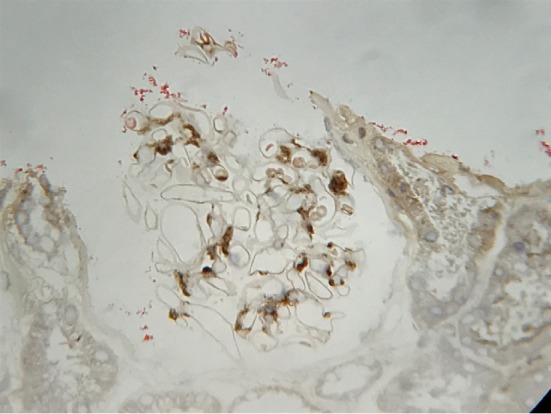
Mesangial C3 deposition was observed.

A 35-year-old Japanese man with a history of renal disease presented to a dermatology clinic with sudden onset of a purpuric rash on his lower extremities (Figure 1) and was diagnosed with anaphylactoid purpura. Although he was asymptomatic, proteinuria, and haematuria were detected during a screening test when he was 20 years old, and he was diagnosed with IgAN based on renal biopsy. Histopathological examination of renal biopsy specimens revealed mild mesangial proliferation (Figure 2). Immunohistochemical examination revealed mesangial IgA (Figure 3), IgG and C3 (Figure 4) depositions, and he was treated with corticosteroids. Thereafter, he moved to our city and continued to be treated here. Corticosteroids treatment was continued for 7 years and was subsequently tapered.

Physical examination following the appearance of purpura revealed purpuric rash without any pitting oedema on his legs. Blood pressure was 147/78 mmHg. Urinalysis showed (+++) urinary protein, no urinary glucose, 6–10 red blood cells/high power field, urinary protein was 1.4 g/g creatinine (Cr). A peripheral blood smear showed a white blood cell (WBC) count of 12,100 cells/mm3, red blood cell of 5.16 million cells/mm3, and platelets 192,000 cells/mm3. Serum haemoglobin was 16.6 g/dL and haematocrit 45.4%. Blood chemistry showed total serum protein 7.2 g/dL, serum albumin 4.2 g/dL, serum sodium 141 mEq/L, potassium 4.3 mEq/L, chloride 105 mEq/L, serum Cr 1.35 mg/dL, serum aspartate aminotransferase 21 IU/L, serum alanine aminotransferase 28 IU/L, blood glucose 96 mg/dL, glycosylated haemoglobin 4.8%, and serum C-reactive protein (CRP) 1.57 mg/dL. Immunological examination showed an antistreptolysin O (ASO) titer 24 IU/mL, IgG 1095 mg/dL, IgA 315 mg/dL, IgM 133 mg/dL, C3 164 mg/dL, C4 35 mg/dL, and CH50 67.0 U/mL. A month before the appearance of purpura urinalysis revealed (++) protein, absence of haematuria, urinary protein level of 0.73 g/gCr, and serum Cr level of 1.10 mg/dL. Two months thereafter, purpura subsided, and his urinary protein level and serum Cr level were restored to the former levels. Skin biopsy was not performed because his purpura was typical for anaphylactoid purpura.

## 3. Discussion

We report a patient who developed anaphylactoid purpura 15 years after the diagnosis of IgAN. Schönlein first described a child with purpura and arthritis in 1837. Subsequently in 1874, Henoch described a patient who showed purpura, arthralgia, and abdominal pain. Therefore, this condition is referred to Henoch-Schönlein purpura. These patients often present with proteinuria and haematuria. Histopathological examination of renal biopsy specimen shows focal segmental mesangial proliferative glomerulonephritis, and mesangial deposition of IgA is a consistent finding. C3 deposition occurs in 90% and IgG deposition in 70% of the cases. The eponym Henoch-Schönlein purpura was replaced with IgAV at the 2012 Chapel Hill Consensus Conference. Based on the revised nomenclature IgAV was defined as vasculitis with IgA1-dominant immune deposits affecting small vessels in the skin and gastrointestinal tract commonly associated with arthritis. IgAV is also associated with glomerulonephritis indistinguishable from IgAN [2]. Currently, it is being recognized that IgAV and IgAN share pathogenic mechanisms. In patients with systemic IgAV or renal-limited IgAN, IgA1 in serum and in tissues deposits shows reduced terminal glycosylation in the hinge regions [3]. Emerging data suggest that patients with IgAV and IgAN have circulating immune complexes containing abnormally glycosylated IgA1 and passively glycan-specific IgG antibodies that form IgA1-IgG and anti-IgA1 immune complexes [4]. IgG antibodies directed against abnormal glycosylation reportedly bind to IgA1 molecules and localize in vessel walls, causing inflammation. Our patient showed increased urinary protein and serum Cr levels coinciding with the appearance of purpura, which indicates a close association between IgAV and IgAN. Notably, the presence of purpura is the only distinguishing feature between IgAV and IgAN. Glomerulonephritis in patients with IgAV is diagnosed on the basis of proteinuria and haematuria and occurs concomitantly with purpura or within a month of onset of purpura. A long interval between the appearance of purpura and the detection of urinary abnormalities is rare [5]. To our knowledge to date, 15 years is the longest interval between the detection of urinary abnormalities and the development of purpura in patients diagnosed with this condition..

The cause of the long interval in our patient remains unknown and could be attributed to the following possibilities: (a) mild purpura could have been present at the time of detection of proteinuria and haematuria. However, considering that the urinary abnormalities were identified during screening, purpura, arthralgia, or abdominal pain was not recognized. Thus, this possibility can be excluded. (b) Previous studies have reported that corticosteroids may effectively treat the extrarenal manifestations in these patients [6] and may inhibit the appearance of purpura. A previous report has described a patient who developed purpura 13 years after being diagnosed with IgAN. Notably, he did not receive corticosteroids [7]. Our patient received corticosteroids for 7 years. Based on these observations, the effect of corticosteroids remains unclear. (c) It is possible that our patient developed an infection at the time of appearance of purpura. Reportedly, 30–50% of patients with IgAV were shown to have streptococcal infection [8]. Although the patient was asymptomatic, his peripheral blood smear showed elevation of the WBC count, and he also showed elevated serum CRP levels suggesting an infection. Infection is a known trigger for vasculitis. However, his ASO titer was within normal limits, and the elevated WBC count and positive results with CRP testing may only represent vasculitis. Diagnosis of IgAV is based on the existence of purpura and is challenging in patients with a long interval between the occurrence of purpura and the diagnosis of IgAN.

## 4. Conclusion

In summary, we describe a rare patient of IgAV who showed purpura 15 years after the diagnosis of IgAN. Therefore, patients with IgAN require close and prolonged follow-up.
